# *Osvaldo *and *Isis *retrotransposons as markers of the *Drosophila buzzatii *colonisation in Australia

**DOI:** 10.1186/1471-2148-11-111

**Published:** 2011-04-24

**Authors:** María Pilar García Guerreiro, Antonio Fontdevila

**Affiliations:** 1 Grup de Biología Evolutiva. Departament de Genètica i Microbiologia, Facultat de Biociències, Universitat Autònoma de Barcelona, 08193 Bellaterra (Barcelona), Spain

## Abstract

**Background:**

Transposable elements (TEs) constitute an important source of genetic variability owing to their jumping and regulatory properties, and are considered to drive species evolution. Several factors that are able to induce TE transposition in genomes have been documented (for example environmental stress and inter- and intra-specific crosses) but in many instances the reasons for TE mobilisation have yet to be elucidated. Colonising populations constitute an ideal model for studying TE behaviour and distribution as they are exposed to different environmental and new demographic conditions. In this study, the distribution of two TEs, *Osvaldo *and *Isis*, was examined in two colonising populations of *D. buzzatii *from Australia. Comparing *Osvaldo *copy numbers between Australian and Old World (reported in previous studies) colonisations provides a valuable tool for elucidating the colonisation process and the effect of new conditions encountered by colonisers on TEs.

**Results:**

The chromosomal distributions of *Osvaldo *and *Isis *retrotransposons in two colonising populations of *D. buzzatii *from Australia revealed sites of high insertion frequency (>10%) and low frequency sites. Comparisons between *Osvaldo *insertion profiles in colonising populations from the Old World and Australia demonstrate a tendency towards a higher number of highly occupied sites with higher insertion frequency in the Old World than in Australian populations. Tests concerning selection against deleterious TE insertions indicate that *Isis *is more controlled by purifying selection than *Osvaldo*. The distribution of both elements on chromosomal arms follows a Poisson distribution and there are non-significant positive correlations between highly occupied sites and chromosomal inversions.

**Conclusions:**

The occupancy profile of *Osvaldo *and *Isis *retrotransposons is characterised by the existence of high and low insertion frequency sites in the populations. These results demonstrate that Australian *D. buzzatii *populations were subjected to a founder effect during the colonisation process. Moreover, there are more sites with high insertion frequency in the Old World colonisation than in the Australian colonisation, indicating a probable stronger bottleneck effect in Australia. The results suggest that selection does not seem to play a major role, compared to demography, in the distribution of transposable elements in the Australian populations.

## Background

Transposable elements (TEs) are DNA sequences that can move along the genome and constitute an important fraction of the genomes of most organisms studied so far [1]. It is indisputable that genomes have evolved in close association with TEs, which are characterised by high species specificity and high structural variability, even in closely related organisms. After several decades of experimental and theoretical work, the mechanisms controlling TE copy numbers in populations and most aspects of their biology within species are still not well understood. Classical theoretical models predict that autonomous elements can be present in the genomes of a population as long as their steady transposition rates can counterbalance their elimination by natural selection [2,3]. This equilibrium hypothesis was considered the principal explanation for the maintenance of a constant TE copy number. However, not all populations are at equilibrium. For instance: first, episodic transposition bursts promote TE genome invasion in natural populations exposed to different population and environmental regimes; second, in other population conditions such as weak selection [4] or migration rates lower than transposition rates [5], it takes a substantial time period to reach equilibrium; third, stress environmental conditions and other population phenomena, including demographic (as in our case) and mating processes, are among the prime causes for the distribution of TEs in genomes. Data concerning plants [6] and nematodes [7] demonstrate that demographic history and mating systems shape TE diversity. However, the exact way in which they contribute to TE genome distribution in *Drosophila *remains obscure, particularly because of the lack of comparative studies on parallel populations submitted to similar environment and/or population conditions. In particular, colonising populations subjected to genetic drift processes due to drastic decreases in population size (i.e. bottlenecks), or confronted by new ecological conditions, could constitute an excellent model for carrying out these studies and evaluate the effect of demography versus selection.

*Drosophila buzzatii*, a cactophilic species that originated in the Chaco region of NW Argentina, colonised the Old World and Australia 300 and 70 years ago, respectively. Whereas the former colonisation was probably associated with South American colonial trade and is not well documented, highly documented information is available for the Australian colonisation. The introduction of *D. buzzatii *into Australia is associated with the program of biological control of the prickly pear (*Opuntia *sp.) infestation that devastated Australian agricultural crops in the 1920s [8]. *D. buzzatii *was most probably introduced into Australia inadvertently via rotten *Opuntia *cladodes containing larvae of *Cactoblastis cactorum*, a natural moth parasite of *Opuntia*, largely being transported in shipments from Argentina, that was used for *Opuntia *pest control. Cactoblastis and the accompanying insects spread rapidly to all *Opuntia*-infested fields [8,9]. After the plague was controlled, *Opuntia *diminished drastically in population size, remaining established in scattered habitat patches [10]. Our previous studies concerning Old World *D. buzzatii *colonisation demonstrated a bimodal pattern of distribution of the *Osvaldo *retrotransposon consisting of high and low insertion frequency sites. However, it is unknown whether this distribution pattern, valid for the Mediterranean colonisation that occurred three centuries ago, is valid for other colonisation processes. The aim of the present work is to study the genome distribution of two retrotransposons, *Osvaldo *[11] and *Isis *[12], in some of these colonising Australian populations of *D. buzzatii*. Subsequently, these results will be compared to those of previous studies concerning the retrotransposon *Osvaldo *in colonising populations of the Iberian Peninsula and in the original Argentina populations in order to evaluate how far TE dynamics depend on population parameters inherent in colonisation processes. Our results show that Australian populations have a mixture of high and low insertion frequency sites for both TEs and the drift effect associated to colonisation is probably the main factor responsible for the TEs distribution frequency. High insertion frequency sites would represent insertions prior to the colonisation. Sites of low frequency would correspond to new insertions occurred after the colonisation.

## Results

### Genomic distribution of *Osvaldo *and *Isis *retrotransposons

**Figure 1:**
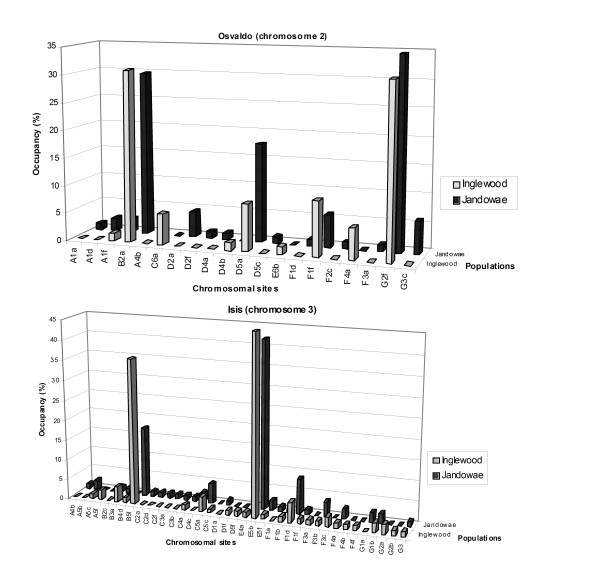
**Distribution of *Osvaldo *and *Isis *on chromosomes 2 and 3 respectively**.

The distribution of *Osvaldo *and *Isis *was analysed in the whole genome of two *D. buzzatii *Australian natural populations. The shape of the chromosomal distribution of these TEs is depicted in Figure 1; *Osvaldo *and *Isis *distributions are represented on chromomes 2 and 3, respectively, as an example. Both TE distributions are characterised by two kinds of sites: sites with low and sites with high (>10%) insertion frequencies. This pattern was not observed for *Osvaldo *on other chromosomes, where the frequency of the highest occupied sites never reached 10% (data not shown). In the case of *Isis*, the same pattern is observed on chromosomes 2 and 5, with occupancy frequencies up to 30% in some sites. *Osvaldo *has three (2B2a, 2D5a and 2G2f) and *Isis *five (2B2a, 2E5a, 3B5f, 3E5b, 5G1a) high occupancy sites in the whole genome. The occupancy (number of times that a site is present in the population) profiles of the two elements are similar in the two populations studied, but *Isis *has a slightly higher occupancy frequency than *Osvaldo *(Table 1). Table 2 summarizes the means and variances of copy number per haploid genome for the two retrotransposons. The mean copy number is low for both elements in the two populations but higher for *Isis *than for *Osvaldo*. Overall mean copy number comparisons between chromosomes demonstrates that whereas the X chromosome presents the lowest mean copy number for both elements, chromosomes 2 and 3 harbor the highest mean numbers of *Osvaldo *and *Isis *copies, respectively. Deviations from Poisson distribution were tested using chi-square goodness of fit, pooling adjacent classes with low expected copy numbers.

**Table 1:** Occupancy profiles of euchromatic sites in Australian colonising populations

TE	Populations	Occupancy profiles																	
		1	2	3	4	5	6	7	8	10	14	15	22	23	25	28	29	34	36
***Osvaldo***	**IN**	37	5	1	3	0	1	1	0	0	0	0	1	1	0	0	0	0	0
	**JA**	33	10	2	1	3	1	0	0	0	0	1	0	0	1	0	1	0	0

***Isis***	**IN**	47	12	1	4	3	3	1	2	2	1	0	0	0	0	1	0	1	0
	**JA**	47	14	7	2	0	1	2	0	0	0	1	0	0	1	0	1	0	1

Population origin: Inglewood (IN) and Jandowae (JA).

Occupancy profiles: number of times that each site is occupied in a population. For example, in the Inglewood population there are 37 sites occupied once, five sites twice, etc.

**Table 2:** Test of the Poisson distribution of *Osvaldo *and *Isis *per chromosome and haploid genome

			Populations						
	**IN (71)**					**JA (84)**			

**TE**	**Ch**.	**m**	**V**_**n**_	**χ**^**2**^	**df**	**m**	**V**_**n**_	**χ**^**2**^	**df**

	**X**	0.08	0.08	-	-	0.07	0,09	3.67	1
	**2**	0.97	0.88	0.79	2	1,14	0,8	2.46	2
***Osvaldo***	**3**	0.25	0.25	0.03	1	0.17	0.24	1.78	1
	**4**	0.20	0.22	0.86	1	0.27	0.22	0.21	1
	**5**	0.18	0.18	0.003	1	0.17	0.14	-	-
	**HG**	1.69	1.96	12.41	3	1.82	1.74	3.99	4

		**IN (80)**				**JA (81)**			

	**Ch**.	**m**	**V**_**n**_	**χ**^**2**^	**df**	**m**	**V**_**n**_	**χ**^**2**^	**df**

	**x**	0,09	0.08	-	-	0.07	0.06	-	-
	**2**	0.75	0.75	1.00	2	0.63	0.44	2.92	1
***Isis***	**3**	1.11	0.94	2.56	3	1.23	0.98	3.03	3
	**4**	0.19	0.18	0.06	1	0.27	0.27	0.26	1
	**5**	0.89	0.56	4.13	1	0.62	0.46	1.31	1
	**HG**	3.02	2.66	3.59	6	2.83	1.57	11.90	6

See Table 1 for population identification. TEs: transposable elements; Ch: chromosome; HG: haploid genome; m: mean copy number; V_n_: variance of copy number; df: degrees of freedom; Bonferroni's correction was applied to the data. *P < 0.05; **P < 0.01. The numbers of individuals analyzed are indicated in parenthesis.

**Figure 2:**
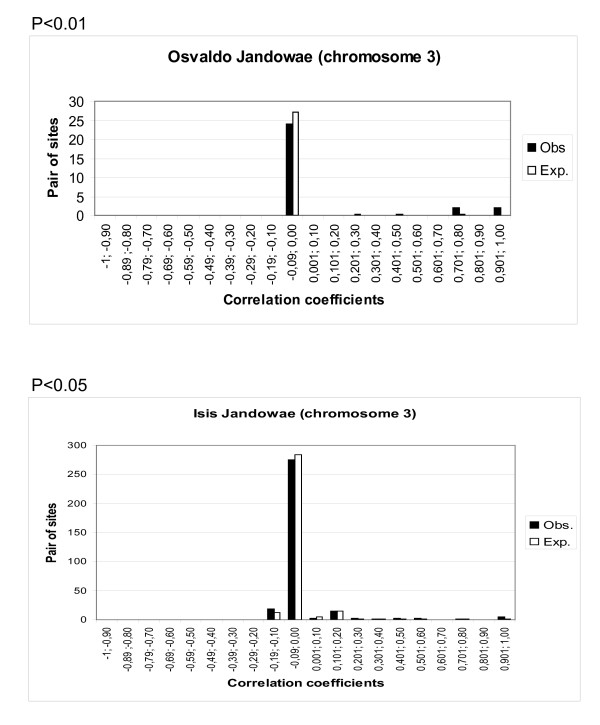
**Observed and expected frequency distributions of correlation coefficients between all pairs of sites of chromosome 3 in Jandowae population**. A) *Osvaldo*; B) *Isis*

In order to test whether the TE insertions are distributed in a repulsive (rarely inserted in nearby regions) or a contagious (frequently inserted in nearby regions) way in each chromosome separately, we computed the linkage disequilibrium for each pair of sites detected by analysing each possible 2 × 2 contingency table [13]. The observed distribution of disequilibria was compared with the expected distribution (assuming no linkage disequilibrium) using Fisher's hypergeometric formula [14]. A battery of correlation tests was performed for each transposable element and chromosomal arm, and the values were pooled at intervals of 0.1. Figure 2 depicts the two tests that produced significant values and represents observed and expected correlation distributions of *Osvaldo *and *Isis *on chromosome 3 of the Jandowae population. Tests were significant for the two elements owing to an overabundance of classes with positive correlation coefficients. In the case of *Isis*, an excess of the class -0.19;-0.10 is observed, but the test is within the limits of significance (P = 0.04)

### Comparative study of *Osvaldo *and *Isis *copy numbers on different chromosomes

The factors controlling TEs in natural populations and the magnitude of their impact on host genomes are topics of much discussion. The genomic distribution of TEs has been interpreted as the result of negative selection against their deleterious effects [3]. Negative selection could act either against deleterious effects of insertions and/or of chromosomal rearrangements resulting from ectopic crossing over between TE copies [2]. If selection plays a preponderant role in TE distribution, fewer insertions should be present on the X chromosome than in autosomes because of the deleterious effects on the X chromosome in hemizygous males [3]. In order to investigate whether this negative selection affected the *Osvaldo *and *Isis *distribution in Australian populations, their copy numbers were compared between chromosome X and autosomes. Observed and expected proportions were compared using a G test [13] among autosomes (Gc), between X and autosomes (Gb) and among all chromosomes (Ga). It was assumed that the expected number of copies was proportional to the relative amount of DNA in each chromosome. The number of bands per chromosome in the cytological map of *D. buzzatii *[15] was considered directly proportional to the amount of DNA, as per previous studies [16,17]. To estimate the expected TE proportions, the proportion of bands of each chromosome was multiplied by the total TE copy number detected in a population. The results of the G test, depicted in Table 3, demonstrate that differences in *Osvaldo *between chromosome X and autosomes are significant when sites of high insertion frequency are considered but not significant when these sites are omitted from the analysis. However, *Isis *appears to be more strongly controlled by natural selection than *Osvaldo *because the results of comparisons between chromosome X and autosomes were significant regardless of whether high insertion sites were considered. This element is characterised by lower and higher copy numbers on chromosomes X and 3, respectively, than would be expected under a random genome distribution. A heterogeneity test [13] was carried out to determine whether the differences always occur in the same direction and the results demonstrate that the two populations generally do not differ for *Isis *and *Osvaldo *copy numbers over the chromosomes. Comparisons among autosomes, when the two populations are pooled and sites of high insertion frequency are eliminated, gave significant results for the *Isis *element but not for *Osvaldo*.

**Table 3:** Comparisons of the proportion of *Osvaldo *and *Isis *among chromosomes

		*Osvaldo*		*Isis*	
**Chromosomes**	**Exp. Prop**.	**IN**	**JA**	**IN**	**JA**

**X**	0.156	0.05	0.04	0.03	0.02
**2**	0.253	0.58	0.63	0.25	0.22
**3**	0.207	0.15	0.09	0.37	0.44
**4**	0.186	0.12	0.15	0.06	0.10
**5**	0.197	0.11	0.09	0,29	0.22

	**Df**				
**Ga**	4	59.44**	130.70**	99.87**	96.09**
		(5.36)	(10.59)	(30.49**)	(32.13**)
**Gb**	1	13.32**	21.53**	42.32**	42.39 **
		(3.88)	(5.54)	(16.49**)	(18.46**)
**Gc**	3	46.12**	82.16**	57.55**	53.70**
		(1.47)	(5.05)	(14.00)	(13.67*)

**Ga**					
Total	8	163.13** (15.95)		195.97**(62.62**)	
Pooled	4	159.94** (13.53**)		189.88**(54.35**)	
H	4	3.19 (2.42)		6.08 (8.27)	
**Gb**					
Total	2	34.85**(9.42*)		84.71** (34.95**)	
Pooled	1	34.67**(9.37**)		84.64** (34.89**)	
H	1	0.18 (0.009)		0.08 (0.06)	
**Gc**					
Total	6	128.28**(6.53)		111.25**(27.67**)	
Pooled	3	125.28**(4.17)		105.25** (19.46**)	
H	3	3.0 (2.36)		6.00(8.21)	

Exp. Prop.: Expected TE proportions; Ga: Comparison of the proportion of TEs among chromosomes; Gb: Comparison of the proportion of TEs between chromosome X and autosomes; Gc: Comparison of the proportion of TEs among autosomes; H: Heterogeneity; Df: degrees of freedom;*P < 0.05; **P < 0.01; Bonferroni's correction was applied. Values of the test excluding high insertion frequency sites are indicated in parenthesis.

### Inversion polymorphism in Australian populations of *D. buzzatii*

The original South American populations of *D. buzzatii *present inversion polymorphisms on chromosomes 2 and 4, with eight (2st, 2j, 2jz^3^, 2jq^7^, 2y^3^, 2jc^9^, 2r^9^, 2js^9^) and two (4s and 4st) chromosome arrangements, respectively [18]. As a result of the colonising processes in Australia and the Old World, rare arrangements were lost and others have changed their frequency. The two Australian populations considered in this study present two arrangements in chromosome 2: st and j, which are also the most frequent in the original and the Old World populations. The frequencies of arrangements st and j are 0.40 and 0.60 in Inglewood and 0.36 and 0.64 in Jandowae, respectively. In contrast to the Old World colonisation, arrangements 2jq^7 ^and 4s have never been detected in Australia [19]. Interestingly, the 2jz3 arrangement, absent in our samples, was identified in other studies at a very low frequency [20].

Recombination has been considered the main factor responsible for the distribution of TEs on chromosomes [21,22]. According to the model proposed by Langley et al. [23], TEs can induce chromosomal rearrangements by ectopic crossing-over between homologous elements located in different chromosomal sites. If the model predictions are true, we will find TE accumulation in regions of low recombination such as inside inversions and adjacent to inversion break-points. In order to determine whether the arrangements are associated with high insertion sites for *Osvaldo *and *Isis*, we computed the product-moment correlation coefficients (r). The results depicted in table 4 demonstrate a significant negative correlation between several highly occupied sites of *Osvaldo *(2D5a and 2G2f) and the j arrangement in Jandowae. Interestingly, in the Inglewood population, correlation coefficients for *Isis *and *Osvaldo *have the same sign (except for the site 2G2f of *Osvaldo*) for both elements, but neither is significant. Moreover, of the sites that have a significant correlation, only one is located inside the j inversion (2D5a); the other is located in its break point (2E5a).

**Table 4:** Correlation coefficients between J chromosomal arrangement and high insertion frequency (HF) sites

		Populations	
	**Arrangement**	**IN**	**JA**

**HF sites of *Osvaldo***		**Corr**.	**Corr**.
2B2a	J	0.02	0.12
2D5a	J	-0.15	-0.30**
2G2f	J	0.08	-0.29**
**HF sites of *Isis***			
2B2a	J	-0.04	-0,21
2E5a	J	0,09	0.02
			

Corr: correlation coefficient. Only sites having a frequency ≥0.10 were considered.

### Comparisons of TE mean copy numbers between Australian and Old World colonisations of *D. buzzatii*

**Figure 3:**
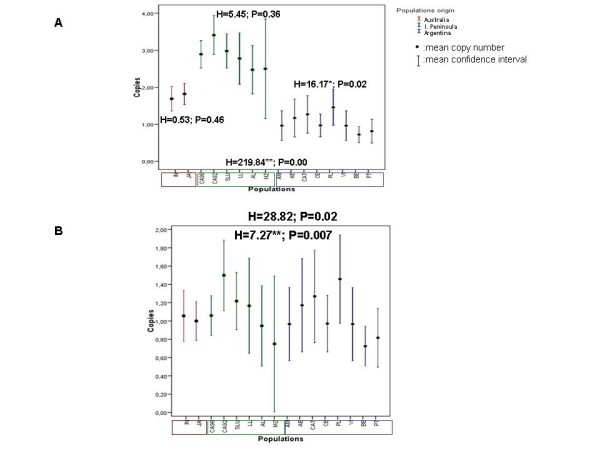
**Kruskal-Wallis non parametric test of analysis of variance for the mean copy number of *Osvaldo***. A) All sites included, B) Excluding high insertion frequency sites. Populations identification: JA (Jandowae), IN(Inglewood), Argentinean and Spanish populations are explained in references [16,17].

The Old World colonisation by *D. buzzatii *has been studied intensively in our laboratory with the *Osvaldo *TE [16,17]. We combined these data with those from the Australia colonisation to compare mean *Osvaldo *copy numbers between both colonising populations and the original ones. Table 5 summarizes the mean *Osvaldo *copy numbers in the natural populations from Argentina and the Iberian Peninsula studied previously, and those from Australia. We observed that the original Argentinean populations have the lowest mean copy number of *Osvaldo*, with a slight difference between the two sets of populations analysed in two previous works [16,17]. Colonising populations from the Iberian Peninsula possessed the highest mean copy number. A Kruskal and Wallis non-parametric test of analysis of variance was performed to investigate whether the mean copy number of *Osvaldo *differs among populations from a given area and between populations from different areas (Figure 3A). While no differences in copy number were observed among Australian populations (H = 0.53, P = 0.46) or among Iberian Peninsula populations (H = 5.45, P = 0.36), there were significant differences among populations from Argentina (H = 16.17*, P = 0.02). When the three population groups were compared, the differences were significant (H = 219.84**, P = 0.00) and the Australian populations had mean copy number values intermediate between the Argentinean and Old World populations. The same analysis was carried out excluding positions of high insertion frequency in the colonising populations (see Figure 3B); the significance disappeared in all cases except when Argentinean and Old World populations were compared (H = 7.27**, P = 0.007). We conclude that the high mean copy number in colonising Australian populations can only be explained by the presence of some sites with high insertion frequency. Colonising populations could have suffered a genomic redistribution of the *Osvaldo *retrotransposon, probably due to the founder effect. As a consequence, certain copies arriving with the founders have a high occupancy in the current populations. These results agree with the hypothesis that the mean copy number of *Osvaldo *increases during the colonisation process. However, in the case of Old World colonising populations, differences still persist after elimination of high insertion frequency sites. The most likely explanation for this is the large mean copy number heterogeneity among Argentinean populations, because when the two populations with the lowest copy numbers are eliminated from the comparison, the differences become insignificant. In the case of *Isis*, a recently characterised element, no information is available from previous population studies, but the mean copy number of laboratory lines from Spanish populations is approximately three copies per genome [12], a value similar to that found in Australian populations.

**Table 5:** Mean copy number of *Osvaldo *retrotransposon in different natural *D. buzzatii *populations: original and colonising

ELEMENT		POPULATIONS			
***Osvaldo***	**Original**		**Colonising**		

	Argentina_a _ (6)	Argentina_b _ (2)	I. Peninsula_a _ (5)	I. Peninsula_b _ (1)	Australia_c _ (2)
					
**Mean**	1.22	0.85	2.85	2.99	1.75

The numbers of populations analyzed are in parenthesis; subscripts represent a detailed explanation about populations and bibliographic references;_a_: Argentinean populations (Amaicha, Arroyo Escobar, Catamarca, Cébila, Palo Labrado and Vipos) and Iberian populations (Carboneras, Sanlúcar, La línea, Mazarrón and Albufeira) as described in Labrador et al. 1998;_b_: Argentinean populations of Berna and Puerto Tirol and Iberian population of Carboneras, as described in García Guerreiro and Fontdevila;_c_: Australian populations (Inglewood and Jandowae) presented in this work.

The sites with the highest insertion frequency of *Osvaldo *in different colonising populations from the Iberian Peninsula and Australia are presented in Table 6. There was a general tendency towards lower numbers of highly occupied *Osvaldo *sites in Australia than in the Iberian Peninsula.

**Table 6:** The highest frequencies of *Osvaldo *insertion sites in two groups of colonising populations

High frequency sites	**I. Península**_**a**_**(6)**	**Australia**_**b**_**(2)**
**2B2a**	0.48	0.31
**2D5a**		0.18
**2D1h**	0.22	
**2F4a**	0.42	
**2F1fg**	0.18	
**2G2f**		0.32
**2G2h**	0.26	
**3F2b**	0.16	
**4C2g**	0.10	
**5A4b**	0.63	

The numbers of populations considered are in parenthesis; subscripts represent bibliographic references:_a_Labrador et al. 1998; García Guerreiro and Fontdevila,_c_this work. See Table 5 for population identification. Only sites having a frequency ≥0.10 are presented.

## Discussion

### TE copy numbers and distribution: Australia *vs*. Old World colonisations

Comparing this study with previous works that analysed original (from Argentina) and colonising (from the Iberian Peninsula and Australia) populations, some differences are apparent. While the original populations always demonstrate low occupancy per chromosomal site, the colonising populations (Australian and Spanish) have some sites with high occupancy. When colonising populations are compared, Old World populations have more highly occupied *Osvaldo *sites (eight) and a higher occupancy rate of some sites (up to 63% occupancy) than the Australian populations (three sites with less than 33% occupancy). The mean copy number is lower in Australian than in Old World populations, placing Australia in an intermediate position between Argentina and the Old World. Data from *Isis *are limited to the Australian populations, but interestingly, the mean copy number is similar in Australian and Old World laboratory stocks.

The overall picture that emerges from these data suggests a global increase in the mean copy number per site of *Osvaldo *during the colonisation process. This may be explained either by the drift effect following the founder event acting on many low occupied sites (turning them into high occupied sites) or by increases of site-directed transposition rates in colonisation, or both. According to previous work [16,17,24,25], sites demonstrating high insertion frequencies are most likely due to a founder effect that occurred during the colonisation process, and low insertion sites are probably the result of new transposition events.

The Australian and Spanish populations have similar insertion-site distribution patterns but some of the aforementioned differences deserve more detailed analysis. First, high insertion frequency sites are generally located in different chromosomal positions in the two colonisations, indicating two independent colonisation events from Argentina. Second, the number of highly occupied sites is greater in the Spanish than in the Australian colonising populations. These differences could be related to specific differences in the two colonising processes. While Old World colonisation appears to be caused by a low number of founders [17], the number of *D. buzzatii *individuals introduced during the Australian colonisation was certainly large initially [8,9] but fell dramatically following the *Opuntia *biological control program. Therefore, we can imagine a scenario where *D. buzzatii *spread rapidly through the *Opuntia *area until 200,000 hectares of prickley-pear had been largely destroyed by biological control [26]. Since *D. buzzatii *is restricted to the *Opuntia *distribution area [27], a parallel drastic decrease of the *D. buzzatii *population size should have occurred. This rapid reduction in the number of coloniser survivors could explain, at least in part, why the number of highly occupied sites in *Osvaldo *is lower in the Australian colonisation than in the Old World one.

The results showed here are in accordance with previous comparisons using allozyme variability [19,28], microsatellites [10,29] and mitochondrial DNA [30], where a decrease in variation was observed in Australian populations, probably due to a bottleneck followed by a population expansion after the initial founder event. Though the founder effect hypothesis is widely accepted by all authors working on Australian *D. buzzatii *populations, there are discrepancies in relation to the bottleneck magnitude depending on the markers used. Whereas nuclear markers such as microsatellites [10] and allozymes [31] suggest a moderate bottleneck, mitochondrial DNA [30] data point to a very low number of founders. The mtDNA was considered a particularly valuable marker for studies of recent population history owing to its maternal inheritance, lack of recombination and rapid evolution (relative to nuclear genes) [32,33]. The fact that data concerning TE distribution tend to be more in agreement with mitochondrial data, and that records of the cactus control program implied a strict quarantine of associated insects, suggest that the most likely explanation is that only a few individuals founded the Australian populations. Therefore, it is expected that the magnitude of the founder effect was stronger in Australian populations than in Old World populations

On the other hand, during colonisation selection could also play un important role for the elimination of deleterious TE insertions. For example, homozygosity can increase during the colonisation process, if a few colonisers are involved in the process, increasing the strength of selection against deleterious insertions as observed in selfing plant species [34,35] Additionally, selection could contribute to the adaptation to new environments as seen during the expansion of *D. melanogaster *from Africa [36,37]. Recent results demonstrate a role for some TEs in the adaptation of *D. melanogaster *colonising populations to the out of Africa temperate climate [38]. It is not impossible that some insertions are maintained because of their positive effects in terms of adaptation to the new environments encountered by colonisers. The bottleneck suffered by Australian populations could have produced gene frequency shifts that have been proposed as responsible for driving populations to new adaptive peaks by selection. But, in the present study, the presence of high insertion sites is more likely to be due to the drift effect associated to the colonisation process because the Australian *D. buzzatii *colonisation time of approximately 840 generations (70 years multiplied by 12 generations per year) is probably insufficient to detect selection effects unequivocally. This hypothesis is supported by results in previous studies where the *Osvaldo *DNA altered sequence and its flanking genomic sequences in high frequency sites were identical in all Old World colonising populations [24], a result highly improbable under the selective hypothesis Thus, when considering all results together we can conclude that selection does not seem to play a major role, compared to demography, in the distribution of transposable elements in the Australian colonising populations.

### TE dynamics in the colonisation processes

Colonisation has been suggested as one of the processes responsible for awakening transposable elements in *D. simulans *[39]. During colonisation, individuals are exposed to stress attributable to new environmental conditions and population regimes. Environmental stresses can induce changes in chromatin structure [1] and promote TE mobilisation as observed in plants [40] and other organisms [41]. *D. buzzatii*, a cactophilic species, has specific resource requirements that imply intense natural selection for survival in extreme environments. Therefore, these species are less able to avoid environmental stress by habitat selection [42].

Considering a very simple model of colonisation, transposition rates in colonisers can be estimated. We assume that sites originating from Argentina during colonisation are found at high frequency in colonising populations and that new transpositions correspond to low frequency sites. The estimations begin with the historical data, which suggest that Australian colonisation of *D. buzzatii *occurred approximately 70 years ago [31,43] and assume a mean of 12 generations per year. Taking the pooled data from the two Australian populations (155 genomes), we estimate 840 generations of colonisation (70 years multiplied by 12 generations), 93 new sites and a mean copy number for *Osvaldo *insertions of 1.75. We estimate a minimum value of transposition rate per site per generation in colonisers, for *Osvaldo*, of 4.08 × 10^-4 ^(93/155 × 1/840 × 1.75) and for *Isis *of 3.62 × 10^-4^. These values have the same order of magnitude as those calculated for *Osvaldo *(1.28 × 10^-4^) in previous studies with natural populations from the Old World [17]. No differences in transposition rates were observed between the two colonisations, or between the two transposable elements considered in this study.

We cannot completely disregard local transposition events in some coloniser populations. It is well known that transpositional bursts have occasionally been found in *Drosophila melanogaster *laboratory stocks [44], other *Drosophila *species [45] and in plants subjected to different environmental stresses [46]. However, transposition bursts are difficult to see in nature in real time because TE copies involved in these events are later silenced owing to their deleterious effect to the host. The majority of these mechanisms include host factors as methylation [47], deletions [48], silencing by RNA interference [49] or random mutations, excisions and purifying selection [50].

In the case of *D. buzzatii *colonisation processes, we can accept that the populations analysed have not suffered large transposition rate changes. Another possibility is to imagine that even if colonisation induced local transposition it could be unnoticed owing to the time elapsed, especially if the TE eliminations are quick, as observed in the *Helena *element in genomes of *D. melanogaster *and *D. simulans *[48]. Old World colonising populations of *D. buzzatii *contain TEs where active and inactive copies coexist [24] and the low occupancy sites observed could be the result of a unique transposition or very few master copies present in the genome. However, the possibility that some low occupied sites harbored copies that have diminished their frequency in populations because of drift or selection effects against insertions with a deleterious effect cannot be disregarded. A colonising population from the Old World (Carboneras) demonstrated a decrease in the insertion frequency of many highly occupied sites (2B2a, 2F4a). In other studies concerning *D. subobscura *colonising species, the existence of common low frequency sites of *bilbo *and *gypsy *elements was attributed to a decrease in insertion frequency in some sites in coloniser populations [25]. However, we hypothesize that high occupancy sites correspond to sites that have increased their frequency by drift associated to the colonisation process. Arguments in favor of this hypothesis are that sites of both elements, found at a low frequency in the original populations, are now at a high frequency in populations resulting from the two colonisations. However, a common highly occupied site (2B2a) of *Osvaldo *and *Isis *elements was observed in the Australian populations. This site could be a transposition hot spot induced by the colonisation process as it is present at a very low frequency in some original populations. Moreover, the two elements are inserted in the same chromosomal band, confirming previous results where *Isis *retrotransposon appeared to have preferential insertions inside *Osvaldo *sequences [12].

Comparisons of TE copy numbers between chromosome X and autosomes, after elimination of high insertion sites, demonstrated that *Isis *appears to be controlled by purifying selection in Australian populations, whereas the selection effect is not so evident for *Osvaldo*. However, the comparison is highly significant when the two Australian populations are pooled, which may suggest a stronger selection intensity against *Osvaldo *insertions in these populations. These differences may be due to a stronger selection effect in the Australian colonisation than the Old World one. Alternatively, the differences observed between the two elements could be due to the existence of differential transposition and regulation mechanisms. Another explanation may be that selection effects on the *Osvaldo *element could go unnoticed if transposition events occurred only a few generations ago. A third possibility is that selection acts more strongly on the *Isis *element (for example, if the element is inserted inside genes or regulatory regions) than on *Osvaldo *and, provided the Australian colonisation is recent, its effects are only detected in the element under the most selection pressure.

Comparisons of the proportion of elements between autosomes demonstrated large differences due to an over-representation of *Osvaldo *on chromosome 2 (58-63% from the total) and of *Isis *on chromosome 3 (37-44% from the total). *D. buzzatii *Australian populations have one inversion on chromosome 2. Inversions [23,51], inversion break-points and nearby regions [52-54] are considered by some authors as sites of accumulation of TEs. These regions are characterised by a reduction in recombination rates that diminishes the probability of deleterious ectopic exchanges. Correlations between highly occupied sites of *Osvaldo *and the J arrangement produce negative values; only two sites were significant: one located outside and another inside the J inversion. The most likely explanation is that most correlations observed are a consequence of the founder effect. This would explain why significant negative correlations are observed even in sites located inside the inversion. A similar interpretation was evident in a study concerning colonising *D. subobscura *populations, where the founder hypothesis was favored by the fact that all correlations between sites and arrangements were significant only in the colonising populations [25]. Moreover, there were positive associations between chromosomal arrangements and highly occupied sites located outside of inversions. This result is not unexpected if we take into account that the effective population size is reduced during the first stages of colonisation. If the TE abundance is regulated by the effect of ectopic recombination between elements [2], the existence of insertions in a homozygous state could reduce ectopic exchange events [35,55] outside inversions, leading to a relaxation of selection pressure.

## Conclusions

Knowledge concerning TE distribution in natural populations, subjected to different environmental and demographic regimes, is an important step towards understanding their role in species evolution. Here we demonstrate that the two retrotransposons studied in Australian populations presented bimodal distribution with high and low insertion frequency sites. These results indicate that Australian *D. buzzatii *populations were subjected to a founder effect and a strong bottleneck during the colonisation process. This work emphasizes the importance of population factors in remodelling the distribution of TEs in the genomes of natural populations.

## Methods

### *Drosophila *strains and mating system

Two natural populations from Southeast Australia: Inglewood (31.55° S/151.52°E) and Jandowae (26.46° S/151.50°E), were caught in March 2006. Of the individuals collected, approximately one hundred males and 15 females of *D. buzzatii *were checked. Immediately after their arrival at the laboratory each wild male was crossed with three virgin females from the control line 63 42/7 F81 [24]. Isofemale lines were established from each wild female and subsequently one F1 male crossed in the same way as described for wild males. The control line was checked by *in situ *hybridisation before the experiment and was characterised as devoid of euchromatic copies of *Osvaldo *bearing two copies of *Isis *element (2F1d and 4F2b), with constant insertion profile over different generations.

Only female larvae were analysed in crosses with the control line in order to have the whole wild haploid genome, including X chromosome. The *Isis *insertion profile of each individual was obtained by subtracting the insertion profile of the control line from that of the F1 larvae. The minor disadvantage of using this method is that, in the case of *Isis*, the putative common sites between wild individuals and the control line were omitted from our studies. However, this is a powerful and reliable method used in previous studies concerning transposable element distribution in *D. buzzatii *and *D. subobscura *[16,17,25]

### DNA probes

A 6.4 kb fragment of the *Osvaldo *retrotransposon, including the main part of the complete element except the two LTRs, was used and cloned in the pBSK vector. The *Isis *probe consisted of a 3.4 kb PCR fragment including pol-env [12] inserted in the pGEM-T vector (Promega). Both probes were labeled with digoxigenin 11d-UTP (Roche) using a random primer reaction.

### *In situ *hybridisation

Polytene chromosome squashes were prepared from *D. buzzatii *salivary glands of third-instar larvae as described by Labrador et al. [56]. Prehybridisation, hybridisation and post-hybridisation washes were performed following a protocol by Roche [57].

*In situ *hybridisation is a reliable technique and the most suitable method for detecting and locating insertions in chromosomal arms. However, the power of resolution of this technique is limited in cases of discrimination of very close sites on the chromosome or elements whose sequence has a divergence below 10%. In the present study, where only a few sites were detected, this is not a drawback.

### Inversions

Inversions were identified after microscopic observation of the same slides used for *in situ *hybridisation. Inversion frequencies were calculated by dividing the number of inversions of each type by the total number of individuals analysed.

### Statistical analyses

Most statistical analyses and graphs were performed using the statistical software SPSS version 15.0 and Excel. In cases of multiple testing, Bonferroni's correction was applied to the data [58].

## Authors' contributions

MPGG participated in the design, the chromosomal slides, in situ hybridisation, statistical analyses and the writing of the manuscript. AF participated in the design, collected Australian populations, directed the project and contributed to writing the manuscript. Both authors read and approved the final manuscript
